# Synthesis and Characterization of Palladium Pincer Bis(carbene) CCC Complexes

**DOI:** 10.1021/acs.organomet.3c00114

**Published:** 2023-04-27

**Authors:** Daniel C. Najera, Gabriel Espinosa Martinez, Alison R. Fout

**Affiliations:** † School of Chemical Sciences, University of Illinois at Urbana-Champaign, Urbana, Illinois 61801, United States; ‡ Department of Chemistry, Texas A&M University, College Station, Texas 77843, United States

## Abstract

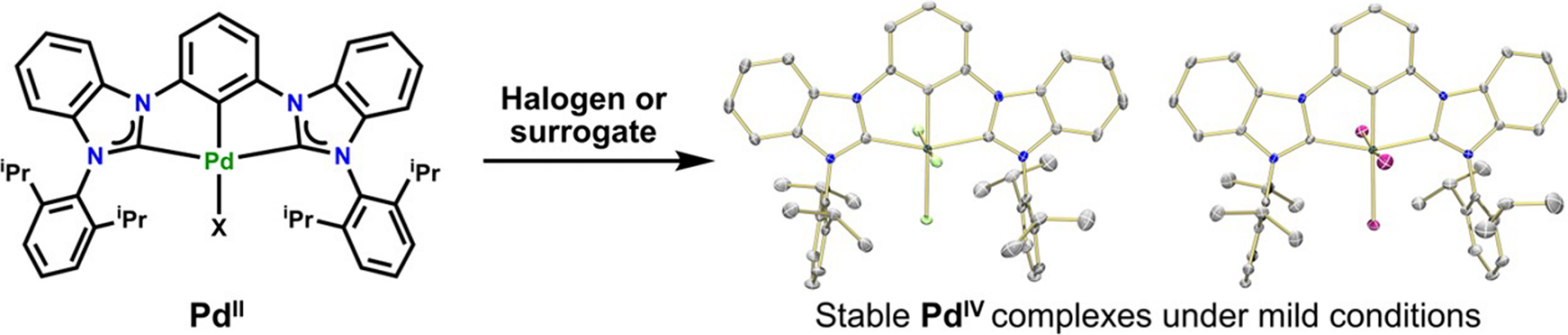

The metalation of the ^DIPP^CCC (^DIPP^CCC = bis(diisopropylphenyl-imidazol-2-ylidene)phenyl) ligand platform with Pd was achieved under mild conditions by reacting [H_3_(^DIPP^CCC)]Cl_2_ with Pd(OAc)_2_ at room temperature in the presence of 3.1 equiv of LiN(SiMe_3_)_2_. The resulting complexes (^DIPP^CCC)PdX (X = Cl or Br) were oxidized by two-electron oxidants PhICl_2_, Br_2_, and BTMABr_3_. All the complexes were crystallographically characterized, and analysis of structural parameters around the ligand scaffold show no evidence of a ligand-centered radical, rendering the metal center in the oxidized species, (^DIPP^CCC)PdX_3_ (X = Cl or Br), a formal Pd^IV^ oxidation state. Unlike their Ni^IV^ analogues, these Pd^IV^ complexes are stable to air and moisture. The addition of styrene to (^DIPP^CCC)PdBr_3_ resulted in the clean reduction of Pd^IV^ to Pd^II^, along with the formation of the halogenated alkane. The oxidation to Pd^IV^ and subsequent return to Pd^II^ upon reduction, as opposed to formation of Pd^III^ species, showcases the accessibility of high-valent palladium ^DIPP^CCC complexes.

## Introduction

Palladium is an essential transition metal in the realm of catalysis and fine chemical synthesis, fostered by predictable and well-established two-electron reactivity. The study of organometallic palladium complexes continues to attract interest, particularly in the context of C–C and C–heteroatom bond forming reactions.^1−3^ Both experimental and theoretical studies on Pd-catalyzed transformations have extensively focused on low-valent Pd^0/II^ systems, while catalytic processes invoking Pd^II/IV^ cycles are a topic of complementary interest toward distinct modes of reactivity.^4−7^ For example, C–halogen bond formation via reductive elimination from Pd^IV^ centers is more thermodynamically favorable than in Pd^II^ to generate halogenated molecules of further utility in organic synthesis^8−12^ and pharmaceutical production.^13,14^ However, stability is a key challenge in the isolation and characterization of high-valent Pd complexes as facile reductive elimination can lead to rapid decomposition. Thus, isolable high-valent palladium species often necessitate the incorporation of multidentate, strongly donating ligands to stabilize the +4 oxidation state.^15,16^

In that regard, pincer ligands usually confer desirable properties for a catalyst on the resulting palladium complex—such as high thermal stability and increased water and air stability—important qualities that make the handling of these compounds relatively easy.^17^ In this class of ligands, Pd complexes incorporating an aryl-linked ECE (E = N, O, S, and P) backbone are some of the most popular.^2^ Ligation of palladium to this class of ligand usually involves the oxidative addition of a Pd^0^ source onto a C–X (X = Br or I) bond.^18−20^ Alternatively, on pincer platforms that feature a C–H instead of a C–X bond, metalations are carried out via C–H activation with a Pd^II^ source.^21−23^ The intermediacy of high-valent Pd pincer complexes in cross-coupling reactions has been proposed for systems where Pd^II^ would be the active catalyst, in contrast with mechanisms in which the Pd^II^ complex serves to dispense catalytically active elemental palladium^24−26^ or those featuring a hemilabile ligand to generate low-ligated species.^27−29^ Still, examples of isolable (ECE)Pd^IV^ remain limited. N-Heterocyclic carbene (NHC) ligands are promising candidates for the study of high-valent Pd complexes. Their synthetic versatility and strong donor capabilities have been shown to stabilize species in high oxidation states.^30−32^ While examples of isolable Pd^IV^ NHC complexes are known, the incorporation of NHCs into ECE ligand scaffolds remains underexplored. Guo and co-workers have described the utility of a monoanionic aryl pincer ligand featuring two mesoionic carbenes (MIC) in the synthesis of isolable Pd^IV^ species that exhibited remarkable air and moisture stability.^33^ Analogously, our investigations into the chemistry of first-row transition metals featuring monoanionic bis(NHC) pincer ligands (CCC) have explored the stabilization of high-valent nickel using a pincer bis(NHC) ^DIPP^CCC (^DIPP^CCC = bis(diisopropylphenyl-imidazol-2-ylidene)phenyl) ligand scaffold. Interested in expanding this effort toward heavier group 10 metals, herein we report the synthesis and characterization of the Pd analogues to the previously reported (^DIPP^CCC)NiX_*n*_ (X = Cl or Br; *n* = 1 or 3)^34^ to explore differences in reactivity between the nickel and palladium complexes.

## Results and Discussion

### Synthesis of (^DIPP^CCC)PdCl (**1**) and (^DIPP^CCC)PdCl_3_ (**2**)

**Scheme 1:**
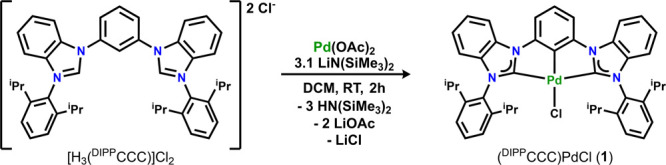
Synthesis of (^DIPP^CCC)PdCl

Initial attempts to metalate the ^DIPP^CCC ligand platform by oxidative addition into the C_Aryl_–H bond by Pd^0^ sources (Pd(PPh_3_)_4_ and Pd_2_(dba)_3_) did not result in the isolation of the expected (^DIPP^CCC)PdH. We turned our attention to metalation via redox-neutral C_Aryl_–H activation with Pd^II^ sources as we previously succeeded in metalating the ligand platform with Ni^II^ sources to form the corresponding (^DIPP^CCC)NiCl.^35^ The reaction of 1 equiv of [H_3_(^DIPP^CCC)]Cl_2_ with PdX_2_ (X = Cl or OAc) and 2.1 equiv of LiN(SiMe_3_)_2_ in dichloromethane at room temperature resulted in the formation of a tan solid after workup. Characterization of the solid resulting from the reaction of either metal starting material by ^1^H NMR spectroscopy revealed the formation of identical products, suggesting that the products bear a chloride ligand. The ^1^H NMR spectrum was consistent with the formation of the desired metal complex, (^DIPP^CCC)PdCl (**1**), as supported by the absence of the corresponding C_Aryl_–H resonance. However, further analysis of the spectrum revealed additional downfield resonances corresponding to a second product with benzimidazolium protons, indicative of incomplete deprotonation of the ligand platform. Optimization of the reaction conditions by employing Pd(OAc)_2_ and 3.1 equiv of LiN(SiMe_3_)_2_ conveniently furnished complex **1** in 84% yield after workup (Scheme 1).

**Figure 1:**
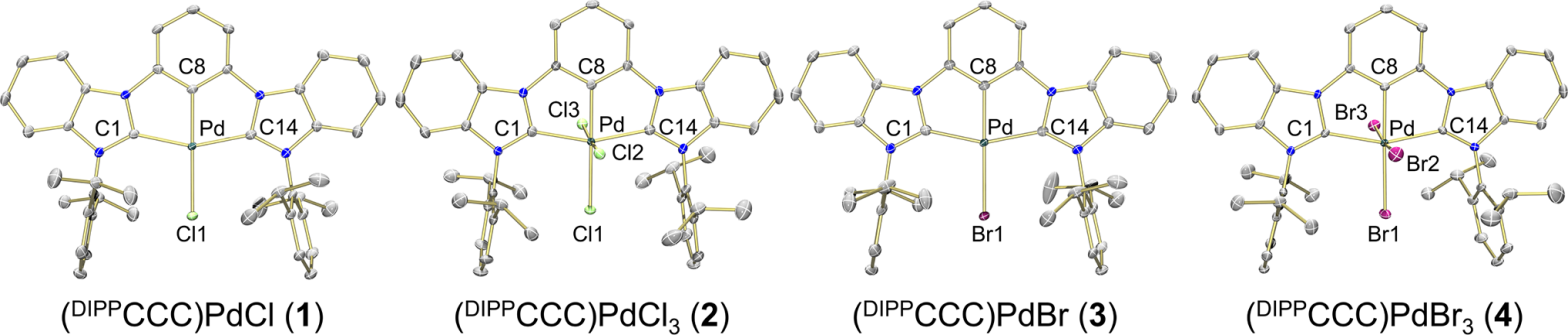
Solid-state structures of **1**, **2**, **3**, and **4** shown with 50% probability ellipsoids. H atoms have been omitted for clarity.

Characterization of **1** by ^1^H NMR spectroscopy revealed a spectrum consistent with the formation of a diamagnetic species (Figure S2), likely monomeric due to the substantial steric profile of the ^DIPP^CCC ligand scaffold. Two doublets integrating to 12H each were located at 1.20 and 0.91 ppm, corresponding to the methyl groups of the flanking diisopropylphenyl substituents on the ligand. Additionally, one septet at 2.40 ppm integrating to 4H signaled *C*_2_-symmetric coordination of the ligand framework. The corresponding aromatic resonances located between 8.09 and 6.96 ppm and integrating to a total of 17H were assigned as the aryl protons of the ligand backbone. Structural characterization of complex **1** via X-ray crystallography confirmed the proposed formulation and the formation of the desired Pd^II^–Cl (Figure 1). Selected bond angles and distances are shown in Table 1. This procedure to ligate Pd into the ^DIPP^CCC ligand platform represents a mild and convenient metalation method compared to the preparation of other Pd pincer complexes through C_Aryl_–H activation of the ligand backbone, which usually requires reflux at temperatures above 100 °C and long reaction times.^36−39^

**Table 1:** Selected Bond Distances and Angles of Complexes **1**–**4**

	**1** (X = Cl)	**2** (X = Cl)	**3** (X = Br)	**4** (X = Br)
Bond distance (Å)				
Pd–C1	2.0463(19)	2.046(4)	2.047(3)	2.075(3)
Pd–C8	1.9509(19)	1.963(4)	1.954(3)	1.965(3)
Pd–C14	2.045(2)	2.048(4)	2.049(3)	2.066(3)
Pd–X1	2.3812(5)	2.3963(10)	2.5121(4)	2.4990(6)
Pd–X2		2.3184(11)		2.4248(5)
Pd–X3		2.3180(11)		2.4760(5)
Bond angles (°)				
C8–Pd–X1	178.85(6)	179.63(13)	178.88(8)	176.27(11)
C1–Pd–C14	157.00(8)	158.80(17)	157.03(12)	158.61(14)
X2–Pd–X3		174.36(4)		174.285(18)

**Scheme 2:**
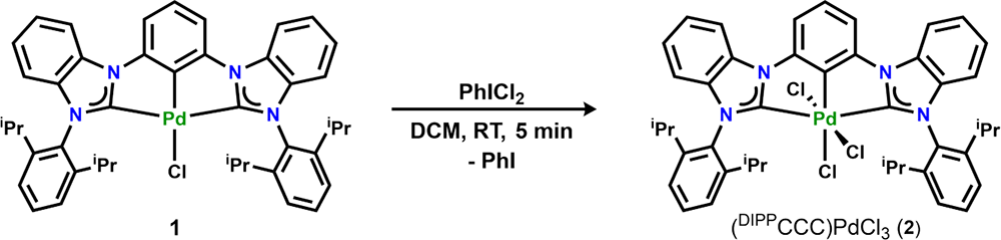
Oxidation of **1** to (^DIPP^CCC)PdCl_3_ (**2**)

Interested in comparing the reactivity of complex **1** with its first-row analogue (^DIPP^CCC)NiCl,^34^ we explored the reactivity of the Pd complex with the two-electron oxidant PhICl_2_ (Scheme 2). The addition of 1 equiv of PhICl_2_ to **1** resulted in an immediate color change from light to bright yellow. Characterization by ^1^H NMR spectroscopy (Figure S4) revealed the formation of a new diamagnetic species, with concomitant formation of PhI. The bright yellow product featured the signature resonances of the ^i^Pr groups at 2.68 (4H), 1.23 (12H), and 0.85 ppm (12H), all still consistent with a *C*_2_-symmetric complex but shifted from the resonances of the starting complex **1**. We assigned the product as (^DIPP^CCC)PdCl_3_ (**2**). Single crystals of **2** suitable for X-ray crystallography revealed the expected octahedral coordination around the Pd^IV^ metal center, featuring two new chloride ligands bound to the metal center.

Comparison of the structural parameters of complexes **1** and **2** showed no significant bond elongation or contraction upon oxidation of the Pd metal center (Table 1). In contrast, the oxidation of (^DIPP^CCC)NiCl to (^DIPP^CCC)NiCl_3_ led to a slight elongation of the ligands around the Ni center, although a contraction was expected due to the increase in charge at the Ni metal center.^34^ We attributed this difference between Ni^II/IV^ and Pd^II/IV^ to the size difference of Ni and Pd. The elongation of the bond lengths around the Ni center was attributed to the change in the coordination environment from square planar to octahedral, requiring additional space for the two new chloride ligands. The larger Pd metal center, however, can accommodate the two new chloride ligands upon oxidation without significantly changing the distances observed in the square planar Pd^II^ complex. Moreover, comparison of bond lengths of the ^DIPP^CCC ligand backbone revealed no significant changes in the ligand platform, precluding the possibility of a ligand-centered radical and rendering the Pd center in complex **2** in the formal +4 oxidation state.

### Synthesis of (^DIPP^CCC)PdBr (**3**) and (^DIPP^CCC)PdBr_3_ (**4**)

Having previously oxidized the (^DIPP^CCC)NiBr complex to (^DIPP^CCC)NiBr_3_ via reaction with elemental bromine, we sought to determine if similar reactivity would occur with Pd. Interestingly, while exchange of the chloride ligand for a bromide ligand in (^DIPP^CCC)NiCl did not proceed via reaction with NaBr in THF, this reaction was successful in the case of complex **1** to obtain complex **3**, (^DIPP^CCC)PdBr, in 92% yield after workup. Characterization of complex **3** by ^1^H NMR spectroscopy revealed two doublets integrating to 12H each located at 1.22 and 0.90 ppm and a septet integrating to 4H at 2.38 ppm corresponding to the ^i^Pr moiety, slightly shifted from the starting complex **1** (1.20, 0.91, and 2.40 ppm, respectively). Additionally, the expected number of resonances was observed in the aromatic region of the ^1^H NMR spectrum (Figure S6).

**Scheme 3:**
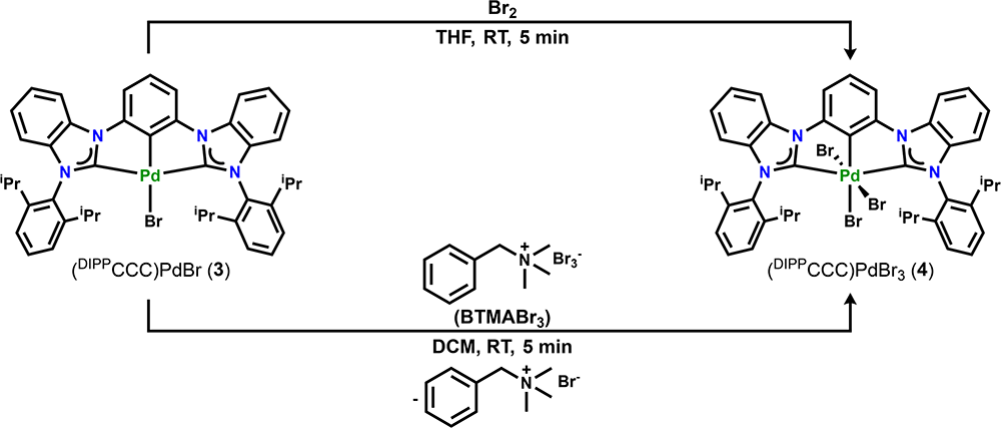
Oxidation of **3** to (^DIPP^CCC)PdBr_3_ (**4**)

Reaction of complex **3** with elemental bromine led to the isolation of an orange solid after workup (Scheme 3). The ^1^H NMR spectrum of the product featured shifted resonances to those of **3**, like the changes in chemical shifts observed upon oxidation of **1** to **2** (Figure S8). The signature doublets of the diisopropylphenyl flanking groups at 1.28 and 0.85 ppm integrating to 12H each and the septet at 2.79 ppm integrating to 4H were shifted from those observed in complex **2** (1.22, 0.90, and 2.38 ppm, respectively). The product was assigned as (^DIPP^CCC)PdBr_3_ (**4**). Both complexes **3** and **4** were crystallographically characterized (Figure 1), and selected bond distances and angles are shown in Table 1.

As observed in the case of the oxidation from **1** to **2**, the oxidation of **3** to **4** did not result in significant bond length changes around the Pd coordination sphere. Moreover, structural parameters of the ^DIPP^CCC platform in **3** and **4** also indicate that there is no radical character in the ligand scaffold. Oxidation of complex **3** to **4** can also be achieved by treatment of **3** with 1 equiv of BTMABr_3_ in dichloromethane (Scheme 3). Although complexes **1**–**4** are not air- or moisture-sensitive, the use of the bromine surrogate BTMABr_3_ as well as the chlorine surrogate PhICl_2_ allow for oxidation of the Pd^II^ complexes in the glovebox without concerns of harming the equipment by using halogens.

**Scheme 4:**
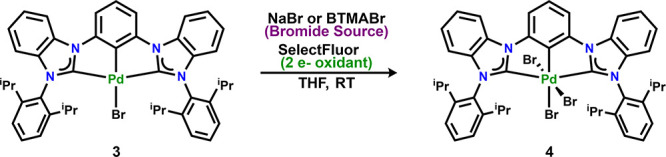
Oxidation of **3** to **4** with SelectFluor

Surprisingly, attempts to isolate Pd^IV^ species with other hypervalent iodide two-electron oxidants was not successful (Ph_2_ICl, PhI(OAc)_2_, and PhI(OOCCF_3_)_2_). These types of reagents, such as the previously mentioned PhICl_2_, are usually powerful enough to oxidize the Pd metal center.^40,41^ We hypothesize that there is a strong preference for halide ligands, hampering the oxidation when other oxidants featuring weakly coordinating anions are employed. The reaction of complexes **1** or **3** with PhI(OAc)_2_ and SelectFluor did not result in the formation of a Pd^IV^ species. However, reaction of **3** in the presence of 2 equiv of Br^–^ (NaBr or BTMABr) followed by the addition of SelectFluor resulted in complete conversion of **3** to **4** (Scheme 4) The observed reactivity was rationalized as proceeding via oxidation of Br^–^ to Br_2_, which then oxidizes the Pd^II^ center to form the expected Pd^IV^ product.

To explore the possible formation of a Pd^III^ complex, **3** was reacted with 0.5 equiv of BTMABr_3_. The ^1^H NMR spectrum of the reaction displayed equimolar amounts of **3** and **4**, with no observable paramagnetic species consistent with the formation of Pd^III^, akin to the results obtained when reacting (^DIPP^CCC)NiBr with 0.5 equiv of BTMABr_3_. The Pd^IV^ complexes **2** and **4** were stable at room temperature for over 48 h in halogenated solvents (dichloromethane and chloroform) and THF and indefinitely in the solid state, with no reduction to Pd^II^ observed. In contrast, (^DIPP^CCC)NiCl_3_ readily degrades in halogenated solvents. Finally, the ability of **4** to transfer Br_2_ to a substrate and cleanly form the reduced Pd^II^ product **3** was assessed by the reaction with excess styrene. Reaction of 1 equiv of **4** with 5 equiv of styrene in CDCl_3_ resulted in complete reduction of **4** to **3** as well as the formation of 1 equiv of (1,2-dibromoethyl)benzene, showcasing similar reactivity to (^DIPP^CCC)NiBr_3_ and to other Pd^IV^ complexes.^33,42^

## Conclusions

In summary, the metalation of the ^DIPP^CCC ligand platform with Pd was achieved under mild conditions by reacting [H_3_(^DIPP^CCC)]Cl_2_ with Pd(OAc)_2_ at room temperature upon the addition of 3.1 equiv of LiN(SiMe_3_)_2_. The complexes (^DIPP^CCC)PdX (X = Cl or Br) were oxidized by two-electron oxidants PhICl_2_, Br_2_, and BTMABr_3_. Although other oxidants suitable for oxidizing Pd^II^ pincer complexes (PhI(OAc)_2_ and SelectFluor) did not result in the expected Pd^IV^ complexes, the addition of a Br^–^ source and a two-electron oxidant still represents a viable way to access high-valent Pd complexes. Compounds **1**–**4** were crystallographically characterized, and analysis of structural parameters around the ligand scaffold shows no evidence of a ligand-centered radical, rendering the metal center in **2** and **4** a formal Pd^IV^ oxidation state. Moreover, the (^DIPP^CCC)Pd^IV^ complexes are more stable than their nickel counterparts, as expected. Finally, addition of styrene resulted in the clean reduction of Pd^IV^ to Pd^II^, along with the formation of the halogenated alkene. The oxidation of **1** and **3** to afford **2** and **4**, respectively, along with the observation of Pd^II^ upon reduction, as opposed to the formation of Pd^III^ species, make (^DIPP^CCC)PdX (X = Cl or Br) great platforms to directly compare the reactivity between nickel and palladium ^DIPP^CCC complexes.

## Experimental Section

### General Considerations

All air- and moisture-sensitive manipulations were performed in an MBraun inert atmosphere drybox under an atmosphere of nitrogen. The MBraun drybox was equipped with two −35 °C freezers for cooling samples and crystallizations. Solvents for sensitive manipulations were dried and deoxygenated on a Glass Contour System (SG Water USA, Nashua, NH) and stored over 4 Å molecular sieves purchased from Strem following literature procedures prior to use.^43^ PdCl_2_, Pd(OAc)_2_, bromine, and styrene were purchased from Sigma-Aldrich and used as received. Benzyltrimethylammonium tribromide (BTMABr_3_) was purchased from Oakwood Chemical and used as received. Lithium hexamethyldisilazide (LiN(SiMe_3_)_2_) was purchased from Sigma-Aldrich and recrystallized from toluene under an inert atmosphere before use. Iodobenzene dichloride (PhICl_2_)^44^ and the ligand [H_3_(^DIPP^CCC)]Cl_2_ (^DIPP^CCC = bis-(diisopropylphenylimidazol 2-ylidene)phenyl)^45^ were prepared according to literature procedures.

NMR solvent (CDCl_3_) was purchased from Cambridge Isotope Laboratories, degassed, and dried with 4 Å molecular sieves. ^1^H and ^13^C NMR spectra were recorded on a Varian spectrometer operating at 500 MHz (^1^H NMR) and 126 MHz (^13^C NMR) at ambient temperature. All chemical shifts were reported relative to the peak of the residual solvent as a standard (CDCl_3_: ^1^H NMR δ *=* 7.26; ^13^C NMR δ *=* 77.16). X-ray analyses were performed at the George L. Clark X-Ray Facility and 3 M Material Laboratory at the University of Illinois at Urbana-Champaign using a Bruker D8 Venture Duo or Bruker X8ApexII diffractometer. Combinations of 0.5° φ and ω scans were used to collect the data. The collection, cell refinement, and integration of intensity data were carried out with the APEX2 software.^46^ Multi-scan absorption correction was performed using SADABS.^47^ The structures were solved with XT^48^ and refined with the full-matrix least-squares SHELXL^49^ program within the Olex2^50^ refinement GUI.

### Preparation of (^DIPP^CCC)PdCl (**1**)

A 20 mL scintillation vial was charged with the benzimidazolium salt [H_3_(^DIPP^CCC)]Cl_2_ (0.050 g, 0.071 mmol), Pd(OAc)_2_ (0.016 g, 0.071 mmol), a stir bar, and approximately 6 mL of DCM. In a separate vial, LiN(SiMe_3_)_2_ (0.037 g, 0.22 mmol) was dissolved in 4 mL of DCM, and this solution was added dropwise to the Pd(OAc)_2_ and benzimidazolium salt mixture. The reaction was stirred for 2 h at room temperature, after which time the solvents were removed under reduced pressure. The resulting solid residue was brought out of the glovebox and suspended in hexanes. The suspension was centrifuged, and the supernatant disposed. After three more rounds of hexanes washes, the resulting solid was taken up in DCM and the resulting mixture was filtered over a pad of Celite. The filtrate was concentrated under reduced pressure to afford a tan solid in good yield (0.0461 g, 0.060 mmol, 84%). Crystals suitable for X-ray diffraction were obtained via vapor diffusion of diethyl ether into a THF solution of the product. Anal. Calcd. for C_44_H_45_ClN_4_Pd: C, 68.48; H, 5.88; N, 7.26. Found C, 68.12; H, 5.52; N, 6.96. NMR data (in CDCl_3_): ^1^H δ = 8.09 (d, *J* = 8.3 Hz, 2H, Ar-C*H*), 7.63 (d, *J* = 8.0 Hz, 2H, Ar-C*H*), 7.52–7.49 (m, 3H, Ar-C*H*), 7.41 (t, *J* = 7.8 Hz, 2H, Ar-C*H*), 7.30 (t, *J* = 7.7 Hz, 2H, Ar-C*H*), 7.22 (d, *J* = 7.8 Hz, 4H, Ar-C*H*), 6.96 (d, *J* = 8.1 Hz, 2H, Ar-C*H*), 2.40 (sept, *J* = 6.9 Hz, 2H, ^i^Pr-C*H*), 1.20 (d, *J* = 6.8 Hz, 12H, ^i^Pr-C*H_3_*), 0.91 (d, *J* = 6.9 Hz, 12H, ^i^Pr C*H_3_*). ^13^C δ = 188.03, 147.43, 147.09, 145.78, 136.94, 131.69, 130.34, 130.27, 125.46, 124.96, 124.04, 123.97, 112.81, 111.64, 109.84, 28.79, 24.23, 24.13.

### Preparation of (^DIPP^CCC)PdCl_3_ (**2**)

A 20 mL scintillation vial was charged with (^DIPP^CCC)PdCl **(1)** (0.025 g, 0.032 mmol), a stir bar, and 3 mL of DCM. To the pale beige solution, PhICl_2_ (9.3 mg, 0.034 mmol) was added, and DCM was used to transfer the oxidant completely. A color change from pale beige to yellow was immediately noted. The reaction was stirred for 5 min, and then, the solvent was removed under reduced pressure. The resulting solid was washed with ether over a pad of Celite and flushed with DCM. Removal of the solvent under reduced pressure afforded the product, (^DIPP^CCC)PdCl_3_**(2)**, as a yellow solid (0.022 g, 0.026 mmol, 81%). Crystals suitable for X-ray analysis were grown by slow evaporation of a solution of **(2)** in DCM. Anal. Calcd. for C_44_H_45_Cl_3_N_4_Pd: C, 62.72; H, 5.39; N, 6.65. Found C, 62.31; H, 5.39; N, 6.27. NMR data (in CDCl_3_): ^1^H δ = 8.19 (d, *J* = 8.3 Hz, 2H, Ar-C*H*), 7.88 (d, *J* = 8.1 Hz, 2H, Ar-C*H*), 7.64 (t, *J* = 8.1 Hz, 1H, Ar-C*H*), 7.60–7.55 (m, 2H, Ar-C*H*), 7.45 (t, *J* = 7.8 Hz, 2H), 7.42–7.38 (m, 2H Ar-C*H*), 7.26 (d, *J* = 7.8 Hz, 4H, Ar-C*H*), 7.09 (d, *J* = 8.2 Hz, 2H, Ar-C*H*), 2.68 (sept, *J* = 6.6 Hz, 4H, ^i^Pr-C*H*), 1.23 (d, *J* = 6.6 Hz, 12H, ^i^Pr-C*H*_3_), 0.85 (d, *J* = 6.9 Hz, 12H, ^i^Pr-C*H*_3_). ^13^C δ = 177.16, 147.04, 146.81, 142.38, 137.09, 131.25, 130.80, 130.29, 127.93, 125.90, 124.96, 124.27, 114.13, 113.18, 112.58, 28.72, 25.73, 24.01.

### Preparation of (^DIPP^CCC)PdBr (**3**)

A 20 mL scintillation vial was charged with (^DIPP^CCC)PdCl **(1)** (0.050 g, 0.065 mmol), NaBr (0.033 g, 0.33 mmol), a stir bar, and approximately 7 mL of THF. The reaction mixture was vigorously stirred overnight at room temperature and filtered over a pad of Celite afterward. The solvent was removed under reduced pressure, and the resulting solid was taken up in DCM and filtered over a pad of Celite. The filtrate was concentrated under reduced pressure to afford the product (^DIPP^CCC)PdBr **(3)** as a tan power (0.049 g, 0.060 mmol, 92%). Crystals suitable for X-ray diffraction were obtained via vapor diffusion of diethyl ether into a THF solution of (^DIPP^CCC)PdBr. Anal. Calcd. for C_44_H_45_BrN_4_Pd: C, 64.75; H, 5.56; N, 6.86. Found C, 64.74; H, 5.52; N, 6.75. NMR data (in CDCl_3_) ^1^H δ = 8.10 (d, *J* = 8.3 Hz, 2H, Ar-C*H*), 7.64 (d, *J* = 8.0 Hz, 2H, Ar-C*H*), 7.53–7.46 (m, 3H), 7.43 (t, *J* = 7.8 Hz, 2H, Ar-C*H*), 7.31 (t, *J* = 7.7 Hz, 2H, Ar-C*H*), 7.23 (d, *J* = 7.8 Hz, 4H, Ar-C*H*), 7.00 (d, *J* = 8.1 Hz, 2H, Ar-C*H*), 2.38 (sept, *J* = 6.8 Hz, 4H, ^i^Pr-C*H*), 1.22 (d, *J* = 6.8 Hz, 12H, ^i^Pr-C*H*_3_), 0.90 (d, *J* = 6.9 Hz, 12H, ^i^Pr-C*H*_3_). ^13^C NMR δ = 187.72, 147.17, 147.14, 145.63, 136.72, 132.10, 130.17, 130.15, 125.47, 124.90, 124.03, 123.93, 112.87, 111.53, 109.78, 28.68, 24.32, 23.

### Preparation of (^DIPP^CCC)PdBr_3_ (**4**)

A 20 mL scintillation vial was charged with (^DIPP^CCC)PdBr (25 mg, 0.031 mmol), a stir bar, and approximately 3 mL of DCM. To this solution, BTMABr_3_ (12.2 mg, 0.063 mmol) was added and the reaction was stirred at room temperature for 5 min. The resulting mixture was filtered through a short silica plug. Removal of dichloromethane under reduced pressure afforded (^DIPP^CCC)PdBr_3_ as an orange solid (28.4 mg, 0.029 mmol, 95%). Crystals suitable for X-ray analysis were obtained via vapor diffusion of hexanes into a benzene/dichloromethane solution of (^DIPP^CCC)PdBr_3_. Anal. Calcd. for C_44_H_45_Br_3_N_4_Pd: C, 54.15; H, 4.65; N, 5.74. Found C, 53.88; H, 4.87; N, 5.43. NMR data (in CDCl_3_) ^1^H δ = 8.21 (d, *J* = 8.3 Hz, 2H, Ar-C*H*), 7.91 (d, *J* = 8.0 Hz, 2H, Ar-C*H*), 7.63–7.55 (m, 3H, Ar-C*H*), 7.45 (t, *J* = 7.8 Hz, 2H, Ar-C*H*), 7.40 (t, *J* = 7.6 Hz, 2H, Ar-C*H*), 7.27 (d, *J* = 8.1 Hz, 95 4H, Ar-C*H*), 7.11 (d, *J* = 7.9 Hz, 2H, Ar-C*H*), 2.79 (sept, *J* = 7.0 Hz, 4H, ^i^Pr-C*H*), 1.28 (d, *J* = 6.7 Hz, 12H, ^i^Pr-C*H*_3_), 0.85 (d, *J* = 6.9 Hz, 12H, ^i^Pr-C*H*_3_). ^13^C NMR (126 MHz, CDCl_3_) δ 174.32, 146.98, 143.07, 142.88, 137.18, 131.43, 130.92, 130.81, 127.69, 125.81, 124.76, 124.45, 114.40, 113.00, 112.53, 77.16, 28.75, 26.00, 24.25.

## Supporting Information Available

Experimental data (reaction of **4** with styrene, ^1^H NMR and ^13^C NMR spectra, crystallographic parameters for **1**–**4**, and selected bond distances and angles for reported complexes) (PDF)
