# Supplementary material for: Synthesis and Characterization of Palladium Pincer Bis(carbene) CCC Complexes
Source: Organometallics. 2023 Apr 27;42(9):832–7. doi: 10.1021/acs.organomet.3c00114 (PMC10863395; doi:10.1021/acs.organomet.3c00114)
Supplement: Supplementary file 1 — om3c00114_si_001.pdf [file om3c00114_si_001.pdf]

Synthesis and Characterization of Palladium Pincer Bis(carbene) CCC Complexes.

*Daniel C. Najera,[a] Gabriel Espinosa-Martinez,[a] Alison R. Fout[b]**

[a] Department of Chemistry, University of Illinois at Urbana-Champaign, Urbana, IL 61801

[b] Department of Chemistry, Texas A&M University, College Station, TX 77840

Supporting Information Table of Contents:

Reaction of 4 with Styrene. 2

NMR Spectra of Metal Complexes 3

Crystallographic Parameters 7

Reaction of 4 with Styrene.

To a 20 mL scintillation vial, **4** (0.010 g, 0.010 mmol) and styrene (0.054 g, 0.052 mmol) were added using $CDCl_3$ as a solvent. After 18 hours, the reaction was monitored by ^1H NMR spectroscopy:

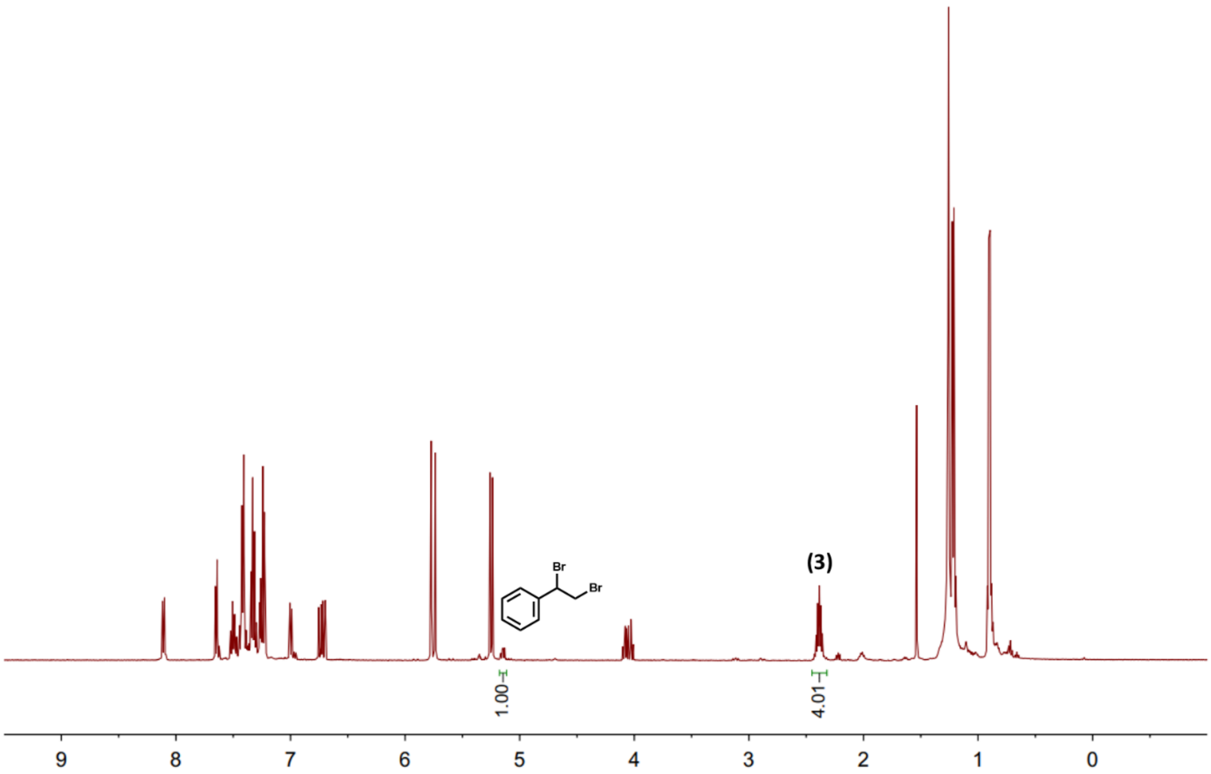

Figure S1. ^1H NMR spectrum ($CDCl_3$, δ = 7.26) of the reaction of **4** with styrene.

NMR Spectra of Metal Complexes

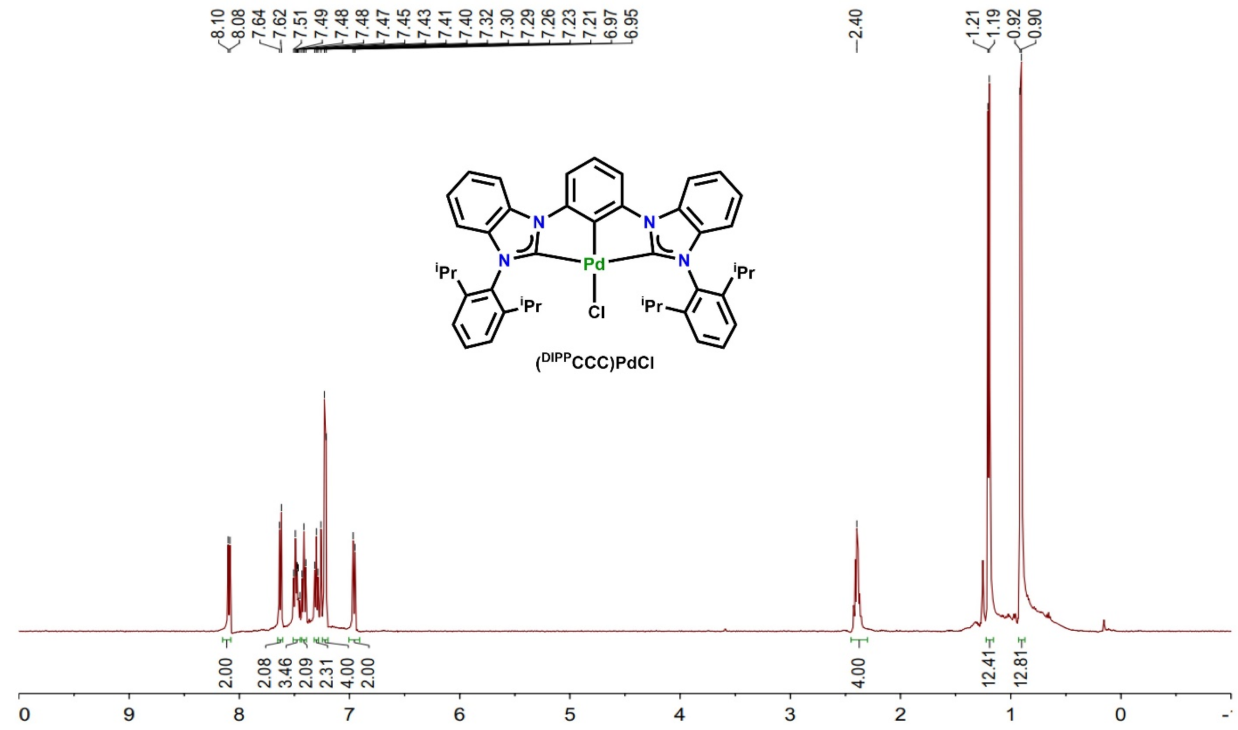

Figure S2. ^{1}H NMR spectrum of (^{DIPP}CCC)PdCl (**1**) ($CDCl_3$**,** δ= 7.26).

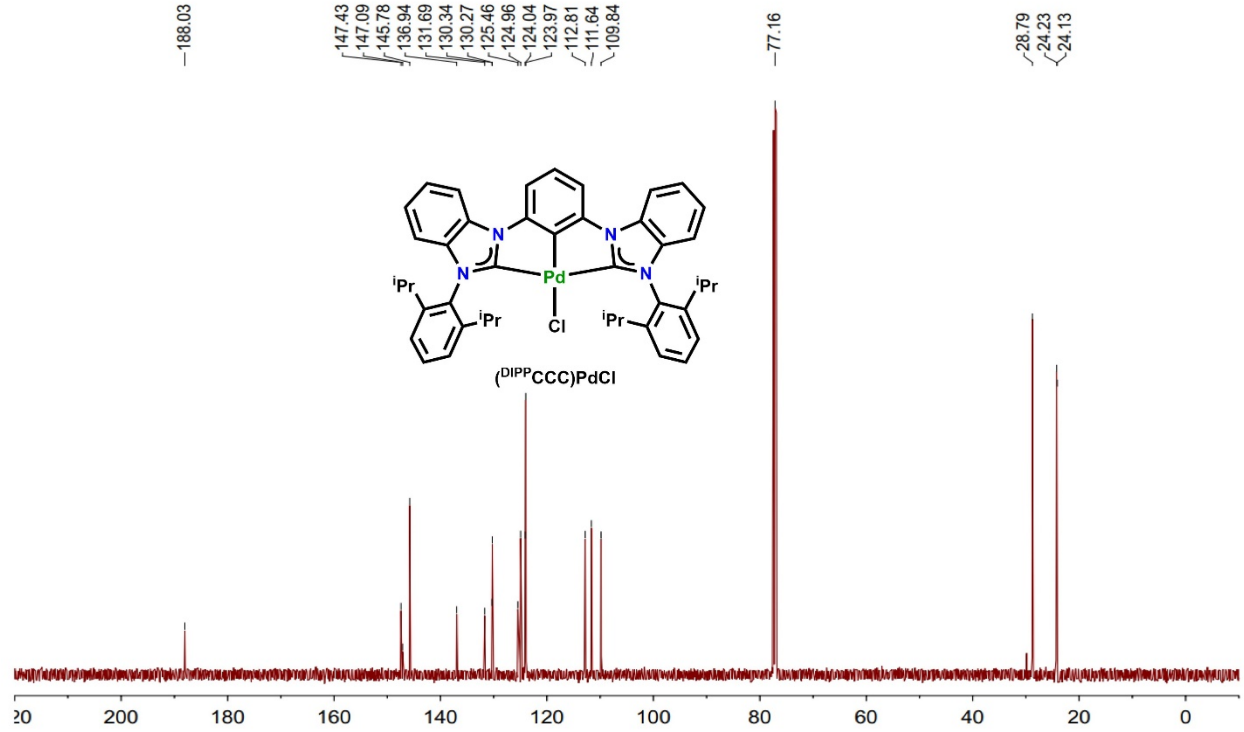

Figure S3. ^{13}C NMR spectrum of (^{DIPP}CCC)PdCl (**1**) ($CDCl_3$**,** δ= 77.16).

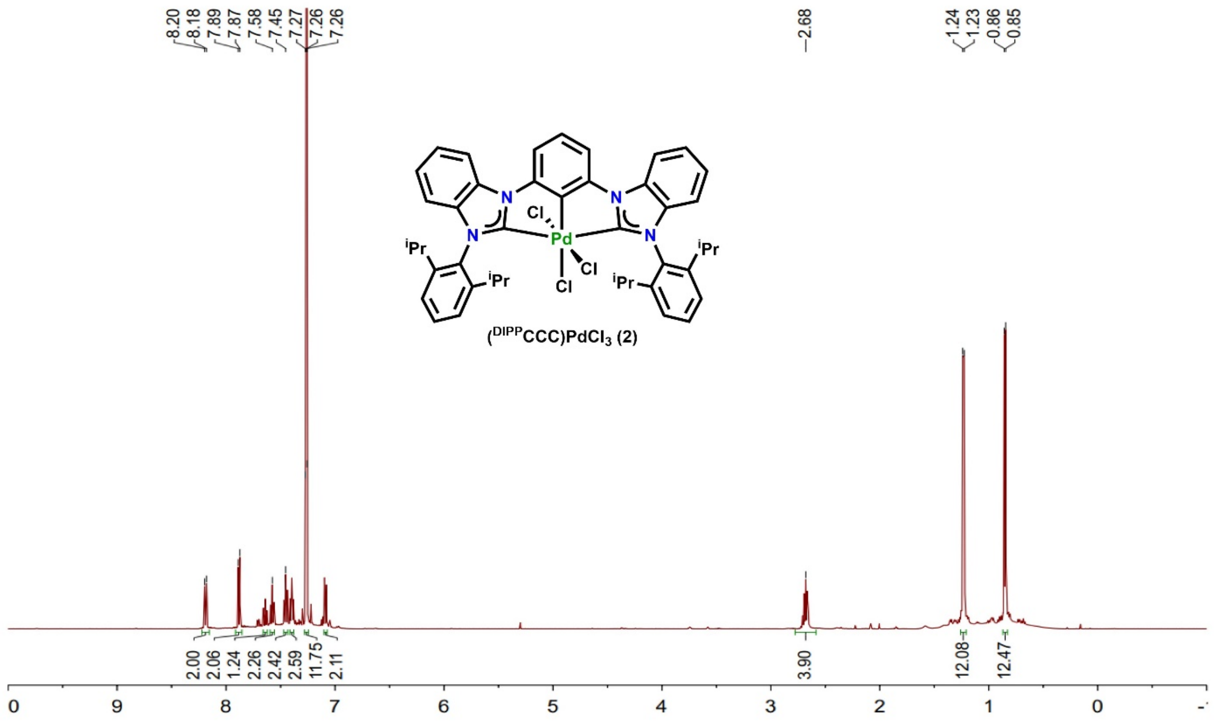

Figure S4. ^{1}H NMR spectrum of (^{DIPP}CCC)PdCl$_3$ (**2**) **(**CDCl$_3$**,** δ= 7.26).

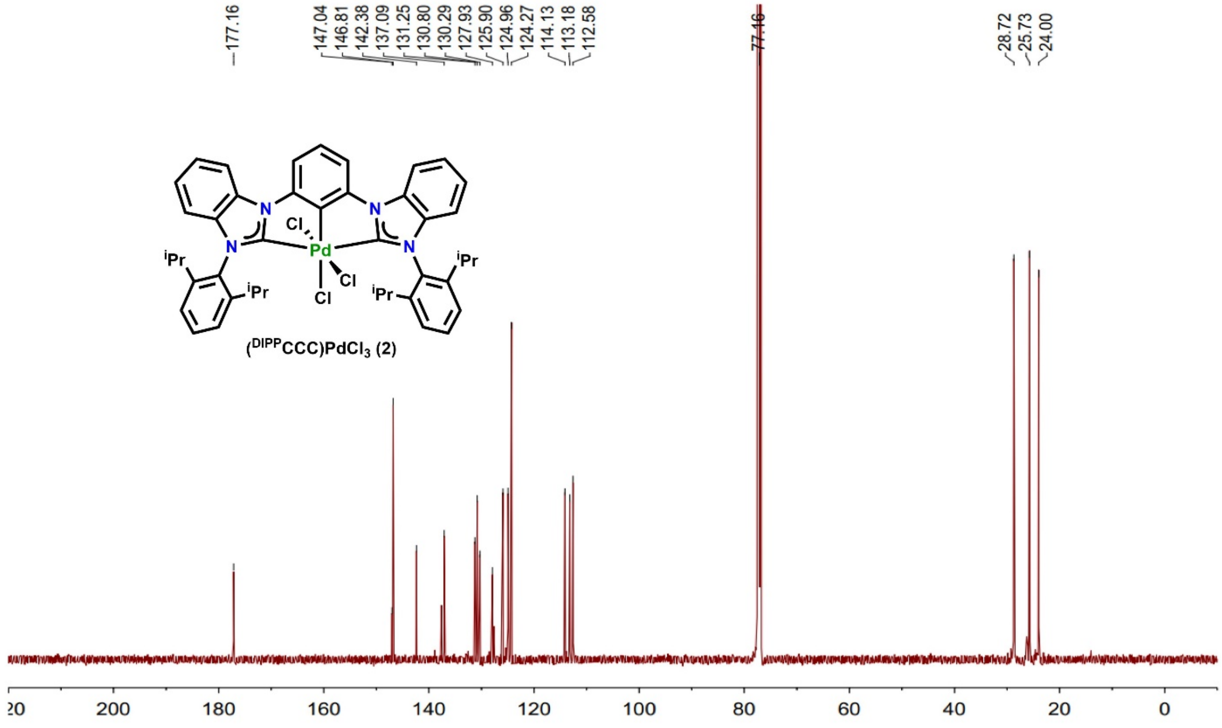

Figure S5. ^{13}C NMR spectrum of (^{DIPP}CCC)PdCl$_3$ (**2**) **(**CDCl$_3$**,** δ= 77.16).

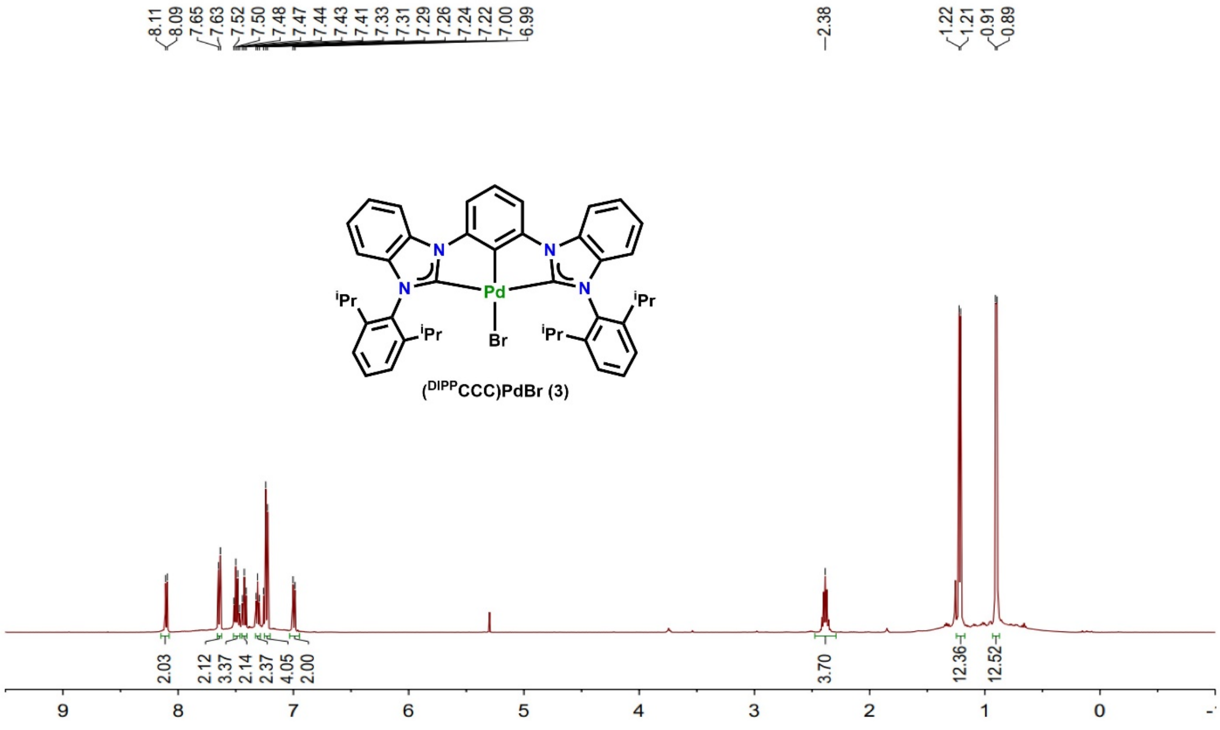

Figure S6. ^{1}H NMR spectrum of (^{DIPP}CCC)PdBr (**3**) **(**CDCl$_3$**,** δ= 7.26).

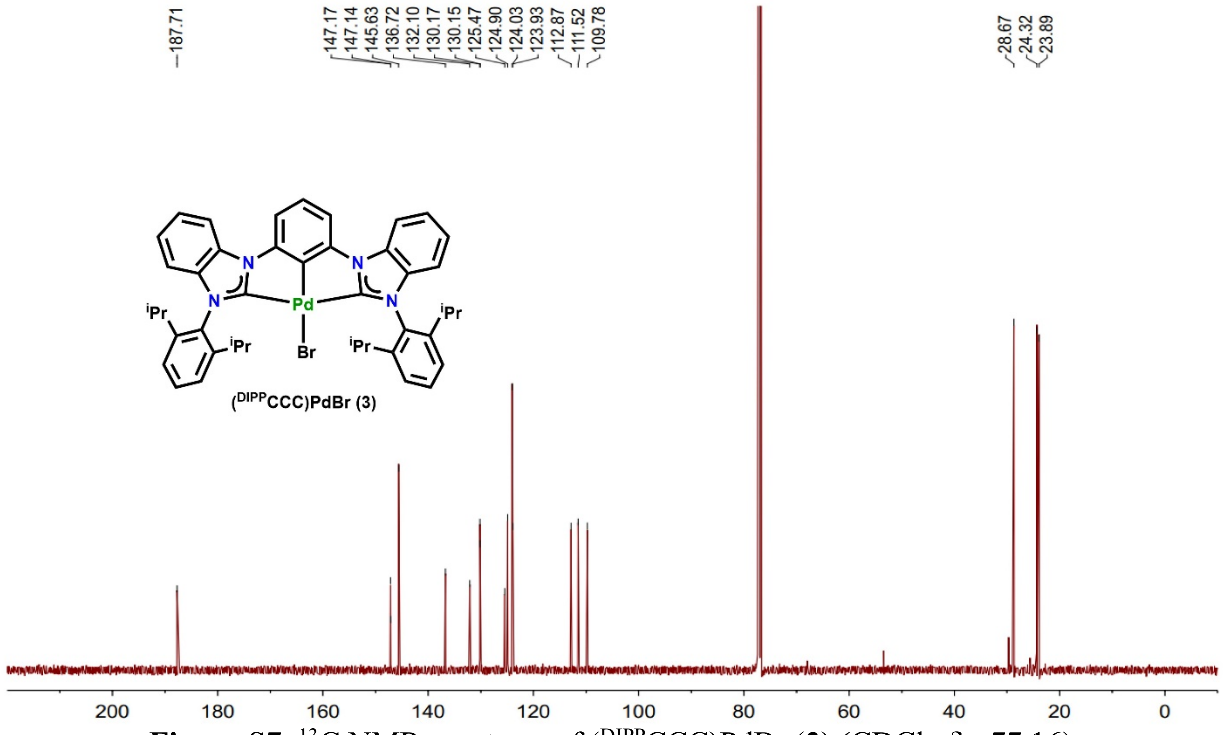

Figure S7. ^{13}C NMR spectrum of (^{DIPP}CCC)PdBr (**3**) **(**CDCl$_3$**,** δ= 77.16).

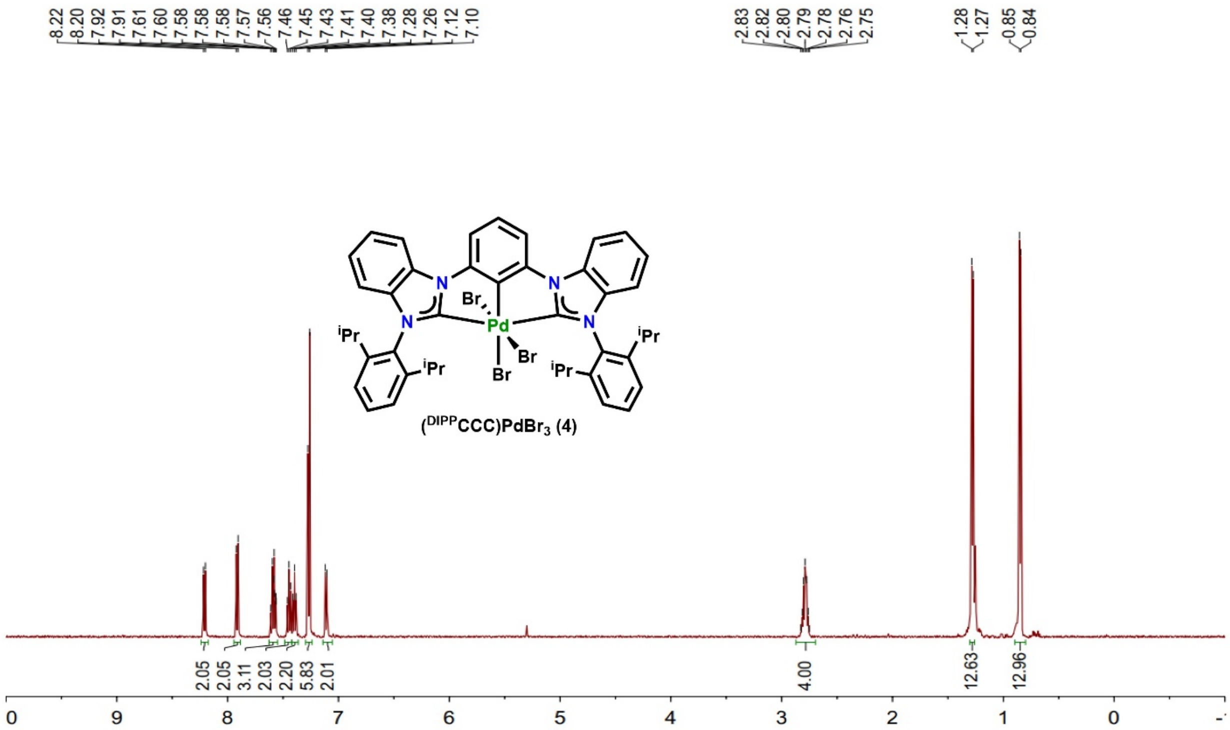

Figure S8. ^{1}H NMR spectrum of (^{DIPP}CCC)PdBr$_3$ (**4**) (CDCl$_3$, δ= 7.26).

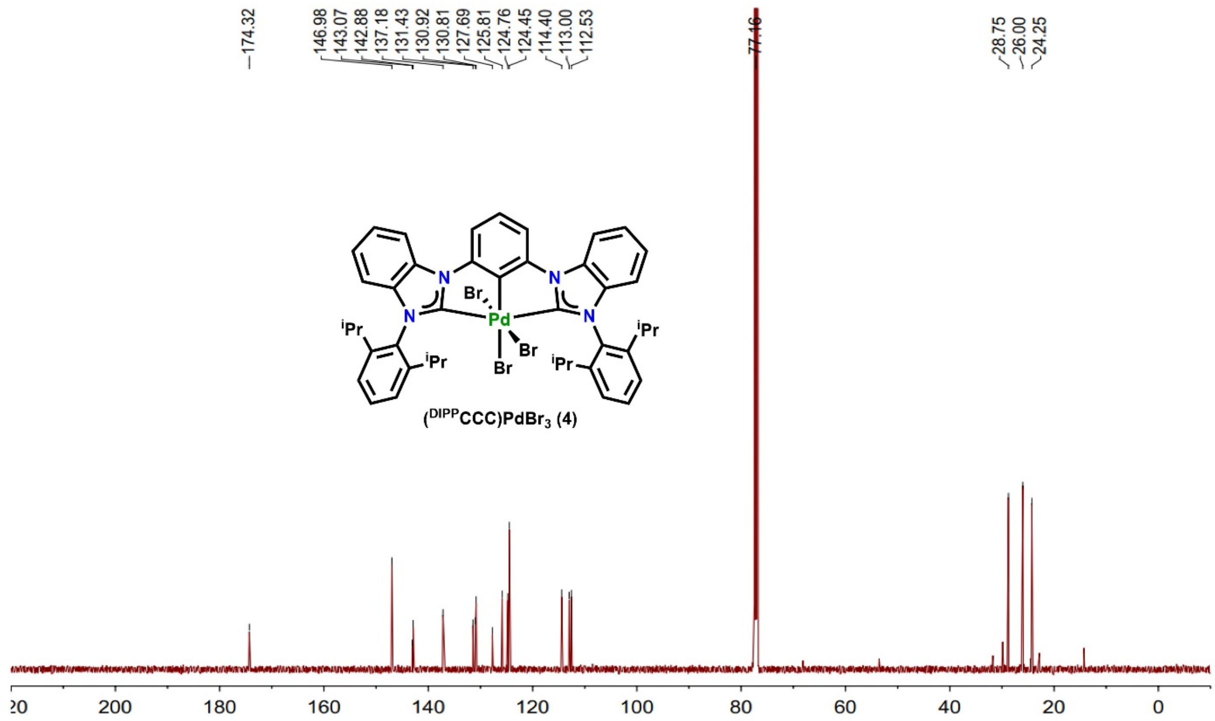

Figure S9. ^{13}C NMR spectrum of (^{DIPP}CCC)PdBr$_3$ (**4**) (CDCl$_3$, δ= 7.26).

Crystallographic Parameters

Table S1. Crystallographic parameters for **1** and **2**.

	(^{DIPP}CCC)PdCl (**1**)	(^{DIPP}CCC)PdCl$_3$ (**2**)
Empirical Formula	$C_{44}H_{45}ClN_4Pd$	$C_{44}H_{45}Cl_3N_4Pd$
Formula Weight	771.69	842.65
Temperature	100 K	100 K
Wavelength	0.71073 Å	0.71073 Å
Crystal system	Monoclinic	Monoclinic
Space group	$P2_1/n$	$P2_1/n$
Unit Cell Dimensions	a = 8.5639(4) Å<br>b = 25.9898(12) Å<br>c = 17.658(1) Å<br>α = 90°<br>β = 92.626(2)°<br>γ = 90°	a = 10.0144(4) Å<br>b = 32.4682(11) Å<br>c = 14.4610(5) Å<br>α = 90°<br>β = 102.5180(10)°<br>γ = 90°
Volume	3926.1(3) Å^3	4590.2(3) Å^3
Z	4	4
Reflections collected	9799	72111
Independent reflections	8718	8440
Goodness-of-fit on F^2	1.102	1.154
Final R indexes [I>=2σ (I)]	R_1 = 0.0355<br>wR_2 = 0.0720	R_1 = 0.0514<br>wR_2 = 0.1207

Table S2. Crystallographic parameters for **3** and **4**.

	(^{DIPP}CCC)PdBr (**3**)	(^{DIPP}CCC)$PdBr_3$ (**4**)
Empirical Formula	$C_{44}H_{45}BrN_4Pd$	$C_{44}H_{45}Br_3N_4Pd$
Formula Weight	816.15	975.97
Temperature	100 K	100 K
Wavelength	0.71073 Å	0.71073 Å
Crystal system	Monoclinic	Monoclinic
Space group	$P2_1/n$	$P2_1/n$
Unit Cell Dimensions	a = 8.5635(5) Å<br>b = 26.0067(15) Å<br>c = 17.7611(10) Å<br>α = 90°<br>β = 92.969(2)°<br>γ = 90°	a = 13.4256(8) Å<br>b = 18.8581(10) Å<br>c = 15.5958(10) Å<br>α = 90°<br>β = 90.936(2)°<br>γ = 90°
Volume	3950.2(4) $Å^3$	3948.0(4) $Å^3$
Z	4	4
Reflections collected	8115	117740
Independent reflections	8115	9811
Goodness-of-fit on F^2	1.153	1.025
Final R indexes [I>=2σ (I)]	R_1 = 0.0391<br>wR_2 = 0.0860	R_1 = 0.0437<br>wR_2 = 0.1111

Table S3. Selected bond distances and angles for reported complexes.

	**1**	**2**	**3**	**4**
**Bond distances (Å)**				
Pd–C_{NHC}	2.0463(19)	2.046(4)	2.047(3)	2.075 (3)
Pd–C_{NHC}	2.045(2)	2.048(4)	2.049(3)	2.066(3)
Pd–C_{Ar}	1.9509(19)	1.963(4)	1.954(3)	1.966(3)
Pd–X1	2.3812(5)	2.3963(10)	2.5121(4)	2.5049(9)
Pd–X2		2.3184(11)		2.4247(5)
Pd–X3		2.3180(11)		2.4760(5)
**Bond angles (°)**				
C_{NHC}–Pd–C_{NHC}	159.38(10)	158.80(17)	157.03(12)	158.61(13)
C_{Ar}–Pd–X1	102.43(11)	179.63(13)	178.88(8)	177.43(10)
X2–Pd–X3		174.36(4)		174.289(17)
